# Small Mutations in *Bordetella pertussis* Are Associated with Selective Sweeps

**DOI:** 10.1371/journal.pone.0046407

**Published:** 2012-09-28

**Authors:** Marjolein van Gent, Marieke J. Bart, Han G. J. van der Heide, Kees J. Heuvelman, Frits R. Mooi

**Affiliations:** Laboratory for Infectious Diseases and Screening, Centre for Infectious Disease Control, National Institute for Public Health and the Environment, Bilthoven, The Netherlands; Universidad Nacional de La Plata, United States of America

## Abstract

*Bordetella pertussis* is the causative agent of pertussis, a highly contagious disease of the human respiratory tract. Despite high vaccination coverage, pertussis has resurged and has become one of the most prevalent vaccine-preventable diseases in developed countries. We have proposed that both waning immunity and pathogen adaptation have contributed to the persistence and resurgence of pertussis. Allelic variation has been found in virulence-associated genes coding for the pertussis toxin A subunit (*ptxA*), pertactin (*prn*), serotype 2 fimbriae (*fim2*), serotype 3 fimbriae (*fim3*) and the promoter for pertussis toxin (*ptxP*). In this study, we investigated how more than 60 years of vaccination has affected the Dutch *B. pertussis* population by combining data from phylogeny, genomics and temporal trends in strain frequencies. Our main focus was on the *ptxA*, *prn*, *fim3* and *ptxP* genes. However, we also compared the genomes of 11 Dutch strains belonging to successful lineages. Our results showed that, between 1949 and 2010, the Dutch *B. pertussis* population has undergone as least four selective sweeps that were associated with small mutations in *ptxA*, *prn*, *fim3* and *ptxP*. Phylogenetic analysis revealed a stepwise adaptation in which mutations accumulated clonally. Genomic analysis revealed a number of additional mutations which may have a contributed to the selective sweeps. Five large deletions were identified which were fixed in the pathogen population. However, only one was linked to a selective sweep. No evidence was found for a role of gene acquisition in pathogen adaptation. Our results suggest that the *B. pertussis* gene repertoire is already well adapted to its current niche and required only fine tuning to persist in the face of vaccination. Further, this work shows that small mutations, even single SNPs, can drive large changes in the populations of bacterial pathogens within a time span of six to 19 years.

## Introduction

Compared to theoretical studies on the effect of vaccination on pathogen evolution [1], [2], [3], [4], there is a paucity of data from field studies, especially with respect to bacterial and protozoan pathogens. There are practical reasons for this, such as the lack of historical strain collections, the relatively complexity bacterial and protozoan genomes and their relative slow evolution rate. The early introduction of vaccination (in the 1940s and 1950s), the presence of extensive strain collections and its clonal and monomorphic nature has made *Bordetella pertussis* an attractive model to study the effect of vaccination on pathogen evolution [1], [3], [5], [6], [7].

The Gram-negative bacterium *B. pertussis* is the main causative agent of whooping cough or pertussis, a highly contagious disease of the human upper respiratory tract (for recent reviews see [6], [8], [9], [10]. Typical symptoms of the disease are paroxysmal coughs which are often followed by vomiting and whooping, due to forced inhalation through restricted airways. Less severe forms of whooping cough are caused by two species which are closely related to *B. pertussis*, *Bordetella parapertussis* and *Bordetella bronchiseptica*. Before childhood vaccination was introduced, pertussis was one of the major causes of infant death worldwide. In The Netherlands, vaccination was introduced in 1953 resulting in a dramatic reduction in pertussis morbidity and mortality [6], [9]. However, despite high vaccine coverage, pertussis resurged in the 1990s in The Netherlands and also in many other countries [6], [10], [11], [12], [13], [14], [15]. Indeed, pertussis has become one of the most prevalent vaccine-preventable diseases in developed countries with estimated infection frequencies of 1–6% [16], [17], [18], [19]. In The Netherlands, the infection frequency was estimated to be 9.3% for the age category >9 years in 2006–2007 [20]. Due to the high circulation rate of *B. pertussis*, adolescents and adults are often the source of infection for newly born who receive their first vaccination at the age of 2–3 months and are therefore unprotected during the first months of life [21], [22].

We have provided evidence that the persistence and resurgence of pertussis in The Netherlands are the compound effect of waning immunity and pathogen adaptation [6], [7], [23]. However, increased awareness and improved detection, may also have contributed to the higher numbers of notified cases. *B. pertussis* is extremely monomorphic [5], [24], [25] and allelic variation was mostly studied in virulence-associated genes, in particular in the genes for the pertussis toxin promoter (*ptxP*), for the pertussis toxin A subunit (*ptxA*), for pertactin (*prn*) and for the serotype 2 and 3 fimbrial subunits (*fim2* and *fim3*, respectively). For *ptxP*, *ptxA*, *prn*, *fim2* and *fim3*, respectively, 18, 8, 13, 2 and 4 alleles have been identified worldwide [6], [26]. In most cases, the differences between the alleles are caused by single nucleotide polymorphisms (SNPs). In the case of *prn*, however, variation is mainly generated by insertions or deletions in a region with repeats, although SNPs have also been identified [6], [23].

Previous work, by us and others, has provided evidence that variation in these genes may have played an important role in adaptation of *B. pertussis* to vaccination [6], [8]. Further, it has been proposed that gene loss may also have contributed to pathogen adaptation, as comparative genomic hybridization using microarrays showed that *B. pertussis* clinical isolates differ in gene content and, over time, a reduction in genome size of *B. pertussis* clinical isolates was observed [27], [28], [29], [30], [31], [32].

Variation in *ptxP*, *ptxA*, *prn*, *fim2* and *fim3* may have an adaptive value as studies in humans and animals have shown that vaccination with these proteins confers protection [6]. Indeed the five proteins are incorporated in different combinations in current acellular pertussis vaccines (ACVs) [9], [10]. Thus variation in these proteins may affect immune recognition and hence strain fitness. Significantly, antigenic divergence has been observed between circulating strains and vaccine strains with respect to these five proteins and several studies in mouse models have provided evidence this affects vaccine efficacy [33], [34], [35], [36], [37]. Besides antigenic divergence between circulating strains and vaccine strains, another adaptive phenomenon may be the recent emergence of strains with increased Ptx production [5], [26], [38], [39]. These strains carry a novel allele for the Ptx promoter, *ptxP3*. The *ptxP3* strains have gradually replaced the resident *ptxP1* strains in the 1990s. Of interest is the close relationship between the emergence of the *ptxP3* strains and increased pertussis notifications in The Netherlands, Finland and Australia [39], [40]. The *ptxp3* strains have probably recently expanded globally [26], [38], [40], [41]. Lately, strains have emerged which do not express genes coding for proteins used in pertussis vaccines, in particular Prn [42], [43], our unpublished data].

The Netherlands offers a number of unique features for the study of the evolution of *B. pertussis*. It comprises a relatively small country, in which vaccination coverage has been consistently high. Further, the vaccines and vaccine strains used have been well characterized, and changes in vaccines and vaccination schedules were implemented nationwide. Mass vaccination against pertussis with a whole cell vaccine (WCV) was introduced in The Netherlands in 1953. The vaccination schedule was initially 3, 4, 5 and 48 months. In 1962, the schedule was changed to 3, 4, 5 and 11 months. Other changes in the vaccination program involved a temporary lowering of the WCV dose between 1975 and 1984, acceleration of the first 3 doses to 2, 3 and 4 months in 1999 instead of 3, 4 and 5 months, the introduction of an ACV booster for 4 year olds in 2001 and, finally, the replacement of the WCV by an ACV in 2005. Two ACVs have been used in The Netherlands, a three component vaccine from GlaxoSmithKline (GSK), containing FHA, Prn and Ptx, and a five component vaccine from Sanofi Pasteur-MSD which contains two additional antigens, Fim2 and Fim3 [9].

Here, we explore how more than sixty years of intensive vaccination has affected the *B. pertussis* population in The Netherlands by integrating data from phylogeny, genomics, and temporal trends in strain frequencies. Our aims were to establish the origin of novel clones, their genetic relationships and to identify novel polymorphic loci potentially involved in adaptation. Our results show that the Dutch *B. pertussis* population has undergone at least four selective sweeps. These sweeps were associated with small mutations, mostly SNPs, in *ptxA*, *ptxP*, *prn* and *fim3*. A number of large deletions were found to be fixed in the *B. pertussis* population, one of which was associated with a selective sweep. Using comparative genomics, additional polymorphism were identified which may have contributed to adaptation of *B. pertussis*. No evidence for a role of gene acquisition in adaptation was found. Our results suggest that small mutations, even single SNPs, can drive large changes in the populations of bacterial pathogens within a time span of six to 19 years.

## Materials and Methods

### Strains

Strains were isolated from Dutch patients that were suspected for pertussis in the period 1949 to 2010. In total 704 strains were used in this study (Supplementary Table S1). Strains were selected randomly, where possible.

### Sequencing of virulence-associated genes

Polymorphisms in the following DNA regions were analyzed: the pertussis toxin promoter (*ptxP*), the gene for the pertussis toxin subunit A (*ptxA*), region 1 of the pertactin gene (*prn*), the genes for serotype 2 (*fim2*) and serotype 3 fimbriae (*fim3*). Amplification and sequencing was performed as described in previous work [23], [39], [44]. A part of the sequence data was derived from previous studies [23], [39], [44]. For DNA isolation, strains were grown on Bordet Gengou (BG) agar plates supplemented with 15% sheep blood and incubated for at 3 to 4 days at 35°C. Bacterial cells were lysed in Tris EDTA buffer (Sigma-Aldrich, Zwijndrecht, Nl, 1.0M Tris-HCl, containing 0.1M EDTA) at 95°C for 5 minutes, centrifuged briefly and used in a PCR. Alternatively, chromosomal DNA was isolated using the GenElute Bacterial Genomic DNA Kit (Sigma-Aldrich, Zwijndrecht, NL) following the manufacturer's instructions for Gram-negative bacteria.

### Single nucleotide polymorphism typing and phylogenetic analysis

The phylogenetic relationship between 198 *B. pertussis* strains, isolated from the period 1949 to 2008, was determined using 85 single nucleotide polymorphisms (SNPs) essentially as described before using the Sequenom technology [41]. Briefly, DNA was amplified by a multiplex PCR followed by single base primer extension reactions. Primer extension products were analyzed using MALDI-TOF mass spectrometry. Supplementary Table S2 contains all SNPs used in this study. The choice of the SNPs was based on the complete genome sequences of the Tohama I strain and six Dutch strains, as described previously [25]. The SNPs were concatenated for each strain resulting in 31 unique sequence types (STs). The concatenated sequences were used to construct a tree with the Maximum Parsimony (MP) algorithm using Bionumerics v6.1 (Applied Maths, Sint-Martens-Latem, Belgium). Bootstrap analysis was performed with 1,000 replicates.

### Diversity index

The diversity index (DI) and 95% confidence interval (CI) of the STs were calculated using the Hunter and Gaston's modification of the Simpson's diversity index [45]. Results were generated using an online tool (V-DICE) provided by the Health Protection Agency's Bioinformatics Unit (available via http://www.hpa.org.uk/).

### Gene loss

Gene loss based on comparative genomic hybridization and genome sequencing was available from previous publications for 65 strains [25], [28]. For this work the genome sequence of an additional five strains was determined to assess gene loss.

### Whole genome sequencing

Five novel genome sequences were derived from Dutch *B. pertussis* isolates, B0296, B0400, B0496, B0738 and B3405, using the Illumina Genome Analyzer system resulting in paired-end reads of 50 base pairs. The sequence data of these strains were submitted to the Sequence Read Archive (SRA) (accession number: SRA051375).

### SNP detection from sequence data

SNPs in 454 data were identified previously [25]. To identify SNPs in Illumina data, reads were mapped to the reference genome *B. pertussis* Tohama I [46] using Maq v0.6.6 [47]. SNPs detected in illumina data were filtered according to the following quality criteria: base call quality >30, mapping quality >30, fraction of reads with SNP>0.90 and read depth >4. The filtered SNP calls from illumina data and 454 data were combined into a single list of SNP loci and the allele of each locus in *B. pertussis* isolate was determined. This allowed recovery of some SNPs that were initially rejected in one isolate because of low confidence but with high confidence in a second isolate. Unmapped reads were mapped to the reference genome *B. parapertussis* 12822 and *B. bronchiseptica* RB50 [46] to identify regions that were not present in *B. pertussis* Tohama I. Information on genes and protein sequences were retrieved from the sequenced genome *B. pertussis* Tohama I. Domain information and conserved positions were recovered from SMART (http://smart.embl-heidelberg.de) and Conserved Domain Database (http://www.ncbi.nlm.nih.gov/Structure/cdd/cdd.shtml). Subcellular localization was predicted by PSORTb version 3.0 (http://www.psort.org/psortb/). Information on uncharacterized proteins was found by BLAST (http://blast.ncbi.nlm.nih.gov/Blast.cgi). Furthermore, a literature search in Pubmed (http://www.ncbi.nlm.nih.gov/pubmed/) was performed to retrieve information on characterized genes.

### Statistical analyses

A Chi-square test was performed to compare the distribution of the three serotypes Fim2^+^, Fim3^+^, Fim2^+^, Fim3^−^ and Fim2^−^, Fim3^+^ in the *fim3-1* population and *fim3-2* population.

## Results

### Genetic relationship between Dutch strains isolated between 1949 and 2008

**Figure 1:**
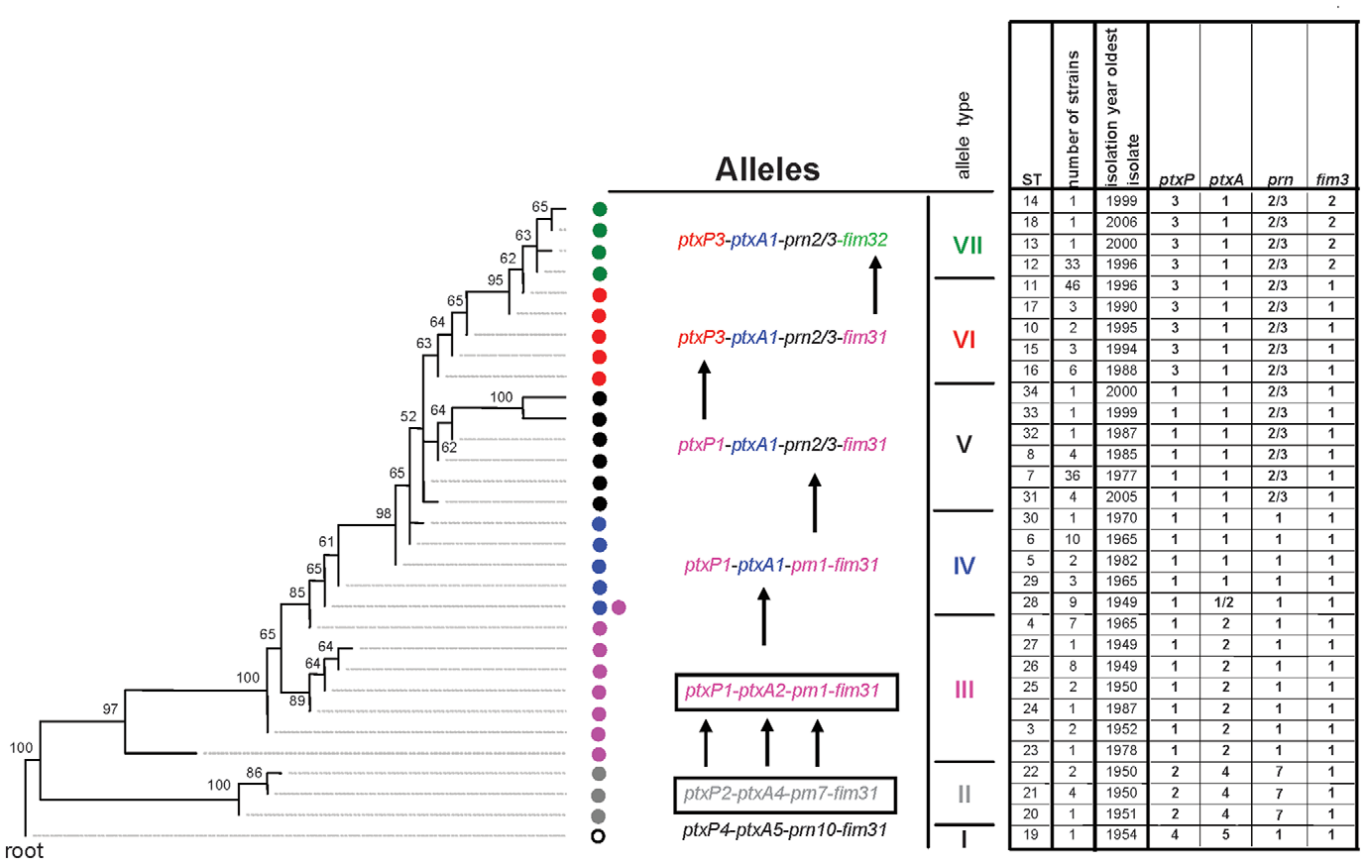
Relationship between phylogeny and the accumulation of mutations in virulence genes. The Maximum Parsimony tree was based on 85 SNPs and 198 Dutch strains isolated between 1949 and 2008. The 85 SNPs resolved the 198 strains in 31 sequence types (STs). Alleles for the pertussis toxin promoter (*ptxP*), the pertussis toxin A subunit (*ptxA*), pertactin (*prn*) and the serotype 3 fimbrial subunit (*fim3*) are indicated. The alleles *prn2* and *prn3* were combined as they are both non-vaccine types. Based on the *ptxP*, *ptxA*, *fim3* and *prn* alleles, seven allele types (ATs), I–VII, could be distinguished. Coloured dots represent distinct ATs and arrows indicate changes between ATs. ATs used for the production of the whole cell and acellular vaccines are blocked. Bootstrap values are indicated in the tree.

The genetic relationship between 198 Dutch strains, isolated between 1949 and 2008, was inferred using the 85 concatenated SNPs and the Maximum Parsimony (MP) algorithm (Fig. 1). Ninety eight Dutch strains were included in a previous study in which various typing methods, including SNP typing, were compared [41]. The tree was rooted with an 18323-like strain (B0442) which is closely related to *B. bronchiseptica*, a species from which *B. pertussis* has evolved [48]. Thirty one sequence types (STs) were identified of which 15 were novel compared to our previous work [41]. Bootstrap values ranged from 52%–100% (average 76%).

The MP tree allowed us to investigate the genetic relationship between strains containing allelic variants of genes implicated in the adaptation of *B. pertussis* [10], [23], [39], [44], [49], [50], [51]: *ptxP*, *ptxA*, *prn*, *fim2* and *fim3* (See Supplementary Fig. S1 for allelic variants used in this study). The *fim2* gene was omitted from this analysis, however, as very little allelic variation was observed in the Dutch population (Supplementary Table S1). Further, alleles for *ptxP*, *ptxA*, *prn* and *fim3*, which were found in low frequencies (≤2%), were included for the calculation of frequencies but are not shown in Fig. 1. Finally, *prn2* and *prn3* were combined, as switching between these alleles may occur with relative high frequency due to slipped strand mispairing in a repeated region.

The genetic relationship between the strains containing allelic variants revealed that the *B. pertussis* population evolved by a successive accumulation of mutations in the four genes. These mutations were found in the trunk of the tree, i.e. were fixed in the population and persisted until replaced by subsequent mutations. Further, there was a notable linkage between particular alleles allowing us to define seven allele types (AT-I to AT-VII), comprised of different allelic combinations of the four genes which were observed in the Dutch *B. pertussis* population (Fig. 1). The seven ATs can also be seen as seven clusters of closely related strains, as defined by the MP tree. Most differences between subsequent ATs were due to a single mutational event, either a point mutation, as in case for *ptxP*, *ptxA* and *fim3*, or the insertion or deletion of a 15 base repeat, as in the case for *prn*. However, the differences between AT-I, AT-II on the one hand and AT-III on the other, involved more than two mutational events. These ATs lie close to the root of the tree, and we presume that our collection did not contain the (hypothetical) intermediates. The two strains used to produce the Dutch WCV belonged to AT-II and AT-III, both of which are found close to the root. The WCV was later replaced by ACVs derived from the 10536 and Tohama I strains, which belonged to, respectively, AT-II and AT-III [52], [53, this work]. When traveling from the root to the tip of the tree, a gradual divergence between the two Dutch WCV strains and the *B. pertussis* population was observed with respect to the four genes. Except for *prn*, which contains three silent SNPs at positions 390, 828 and 831, all mutations in ORFs resulted in amino acid changes (Supplementary Fig. S1).

Looking within particular ATs, it was clear that certain STs predominated. Of particular note are ST-7, ST-11 and ST12, which represent 76%, 76% and 92% of the strains within, respectively, AT-V, AT-VI and AT-VII (Fig. 1). Assuming this was not due to a sampling artefact, this suggests that other, as yet unidentified, mutations are responsible for fitness differences within ATs.

### Temporal trends in AT frequencies in The Netherlands in the period 1949–2010

**Figure 2:**
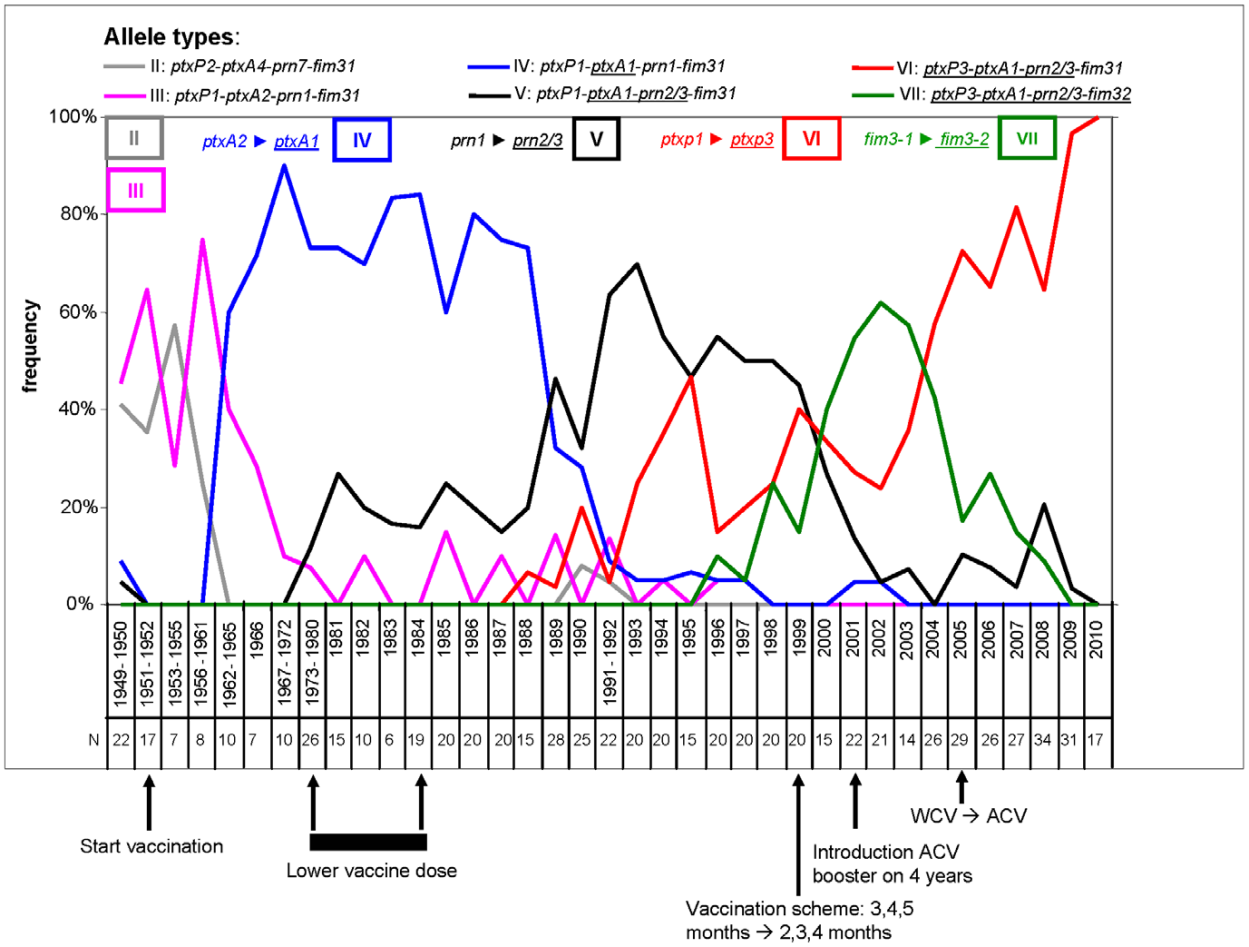
Temporal trends in strain frequencies and notifications in The Netherlands in the period 1949–2010. Strain frequencies are indicated by coloured lines. Strains were aggregated into allele types (ATs) defined by the combination of alleles for *ptxP*, *ptxA*, *prn* and *fim3* as shown in the top of the graph. No distinction was made between strains with the *prn2* and *prn3* alleles. ATs are indicated by blocked Roman numbers and allele changes resulting in differences between ATs are indicated. Non-vaccine type alleles are underlined. ATs found in one or two periods only, with a frequency lower than 15%, are not shown. If necessary, years were combined to increase the number of analyzed strains to at least 6. Note that due to this, the X-axis is not proportional. Changes in the vaccination program are indicated below the X-axis. From 1962 to 1999, four doses of WCV were given at the ages 3, 4, 5 and 11 months. In 1999, the schedule was changed to 2, 3, 4 and 11 months. In 2001, a booster with an ACV was introduced for 4 year olds and in 2005 the WCV was replaced by an ACV for all age categories. Between 1975 and 1984, the WCV dose was temporarily reduced. Abbreviations: N, number of strains analysed; WCV, whole cell vaccine; ACV, acellular vaccine.

Next, we explored the temporal trends in AT frequencies in The Netherlands in the period 1949–2010 (Fig. 2). For this, we determined the ATs for an additional 506 Dutch strains isolated between 1949 and 2010, resulting in a total of 704 strains for which the AT was known. This is an extension and update of previous work [23], [39], [44], [54].

Before the introduction of vaccination in 1953, AT-II and AT-III predominated. As mentioned before, the strains used for production of the Dutch WCV and ACVs, belong to these ATs. Approximately ten years after the introduction of vaccination, AT-IV emerged which differed from AT-III by a non-silent point mutation in the *ptxA* gene (allele designation, *ptxA1*). This mutation resulted in a mismatch with the vaccine strains. AT-IV nearly completely replaced AT-II and AT-III, representing 60% to 90% of all isolates in the period 1962 to 1988. AT-IV was subsequently gradually replaced by AT-V. AT-IV and AT-V differ with respect to the *prn* alleles. While AT-IV contained the vaccine type allele *prn1*, AT-V harboured either *prn2* or *prn3*. The three *prn* alleles code for three different protein variants. AT-V was first detected in the period 1973–1980 (frequency 11%), and reached high frequencies in the period 1991 to 1998 (frequency 47%-70%). AT-V was replaced by AT-VI and AT-VII. While AT-V contained *ptxP1*, AT-VI and AT-VII contained a novel promoter allele, designated *ptxP3*. Strains with the *ptxP3* allele were first detected in 1988 and since 2001 more than 90% of the *B. pertussis* population was comprised of *ptxP3* strains. Strains with the *ptxP3* allele were associated with two *fim3* alleles, *fim3-1* (the vaccine type) and *fim3-2*, designated as AT-VI and AT-VII, respectively. AT-VII was first detected in 1996, increased in frequency to 62% in 2002 and then decreased in frequency. Presently, 100% of the *B. pertussis* populations consist of AT-VI.

The rise and fall in AT-VII (and hence *fim3-2*) frequencies, suggested frequency-dependent selection. If this assumption is true, it suggests that one amino acid change can cause a significant shift in the *B. pertussis* population in a few years although we cannot exclude a role for other as yet unidentified polymorphic loci. We explored this issue further by determining the frequency with which the *fim3-2* allele was expressed. The *fim2* and *fim3* genes are subject to phase variation and switched on and off by insertions and deletions in a homopolymeric C-stretch in the promoter region [55]. Thus strains may express both fimbrial genes (i.e. carry the phenotype Fim2^+^, Fim3^+^), or more generally, express either *fim2* or *fim3* (resulting in the phenotypes Fim2^+^, Fim3^−^ and Fim2^−^, Fim3^+^, respectively). If the switch from *fim3-1* to *fim3-2* has driven the expansion of AT-VII, one would expect *fim3-2* to be mainly associated with strains expressing *fim3*. This is indeed what we found. In the period 1995–2008, when *fim3-2* was detected in the Dutch *B. pertussis* population, 99% of the *fim3-2* strains expressed *fim3* (N 74) (P<0.0001). Only 1% of the Fim2^+^ Fim3^−^ strains (N 81) contained a (silent) *fim3-*2 allele.

### Genetic diversity

**Figure 3:**
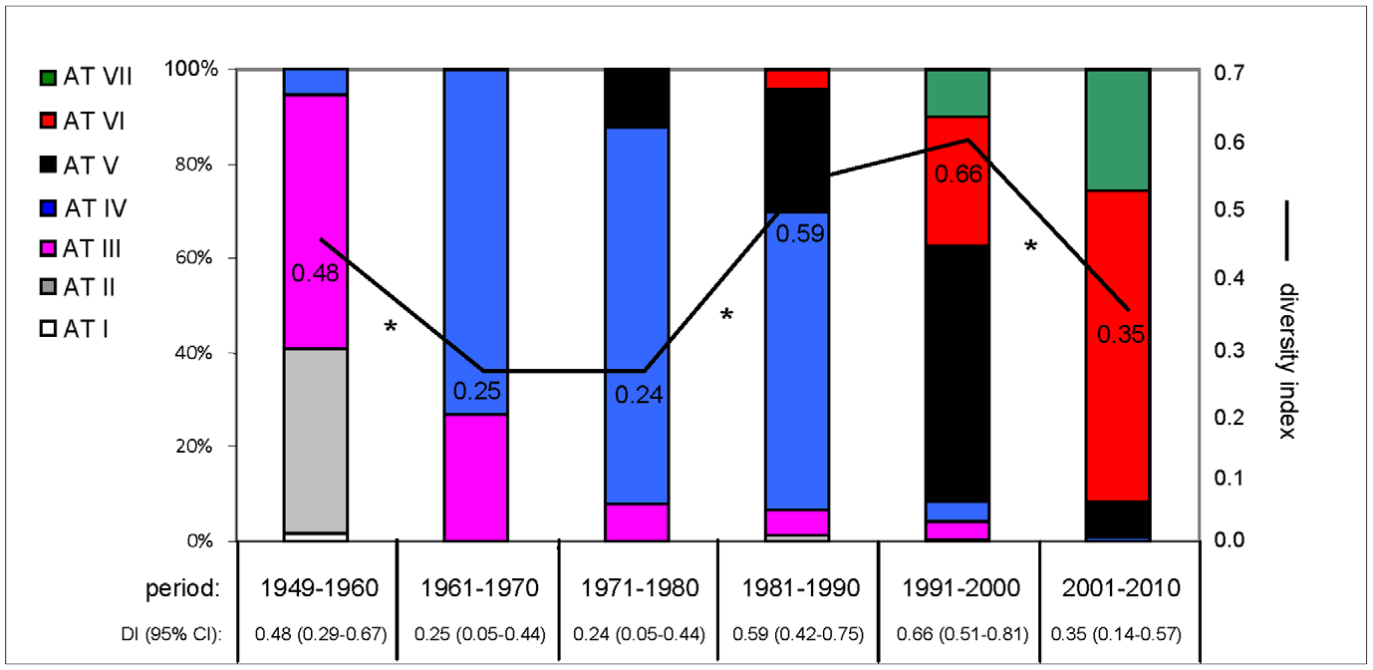
Frequency of allele types and genetic diversity of the Dutch *B. pertussis* population in the period 1949–2010. The diversity index (DI) and 95% confidence interval (CI) for each period, based on the sequence types (STs), were calculated using the Hunter and Gaston's modification (Hunter and Gaston 1988) of the Simpson's diversity index and are indicated. Frequencies of allele types are calculated for each period and coloured as in Fig. 1. Significant differences in DI between periods are indicated with asterisks. Abbreviations: AT, allele type; N, number of strains; DI, diversity index; CI, 95% confidence interval.

Decreases and increases in genetic diversity may reflect clonal expansion and strain diversification, respectively. Using the frequency of STs, we explored the relationship between variations in genetic diversity and AT frequencies (Fig. 3). For this, the diversity index (DI) based on the 31 STs was calculated for periods of approximately 10 years. Between the periods 1949–1960 and 1961–1970, AT-IV expanded, largely replacing AT-II and AT-III. AT-IV differs from AT-II and AT-III in all four alleles and *ptxA* only, respectively. The expansion of AT-IV coincided with a decrease in DI from 0.48 to 0.25. The following two periods, 1961–1970 and 1971–1980, revealed only a slight increase in AT-IV frequency, while the DI was slightly reduced from 0.25 to 0.24. Between 1971–1980 and 1981–1990 AT-IV decreased slightly in frequency while AT-V emerged. Between these periods an increase in DI was observed from 0.24 to 0.59. This may reflect diversification of AT-IV, which predominated for 27 years from 1962–1988. In the period 1991–2000, AT-IV was reduced in frequency and mainly replaced by AT-V, although AT-VI and AT-VII also increased in frequency compared to the previous period. AT-IV and AT-V differ in *prn* alleles only, containing respectively *prn1* and *prn2* or *prn3*. Differences between these three alleles occur by deletions and insertion of 15 base repeats. Such mutations occur with higher frequencies than point mutations, possibly allowing a more polyclonal expansion and explaining why DI did not decrease between 1981–1990 and 1991–2000, but actually increased from 0.59 to 0.66. In the last two periods, 1991–2000 and 2001–2010 a significant expansion was observed of AT-VI and to a lesser extent of AT-VII. These expansions coincided with a decrease in DI from 0.66 to 0.35. The difference between AT-V and AT-VI is a single point mutation in *ptxP*, while AT-VII contains an additional mutation in *fim3*.

In summary, changes in alleles due to SNPs were associated with decreases in genetic diversity in the bacterial population, suggesting clonal expansions. In contrast, changes in alleles due to variation in repeats were associated with a (slight) increase in genetic diversity, suggesting a polyclonal expansion. These observations are consistent with the relative frequency in which point mutations and mutations in repeated regions occur.

### The role of gene loss in adaptation of *B. pertussis*

**Figure 4:**
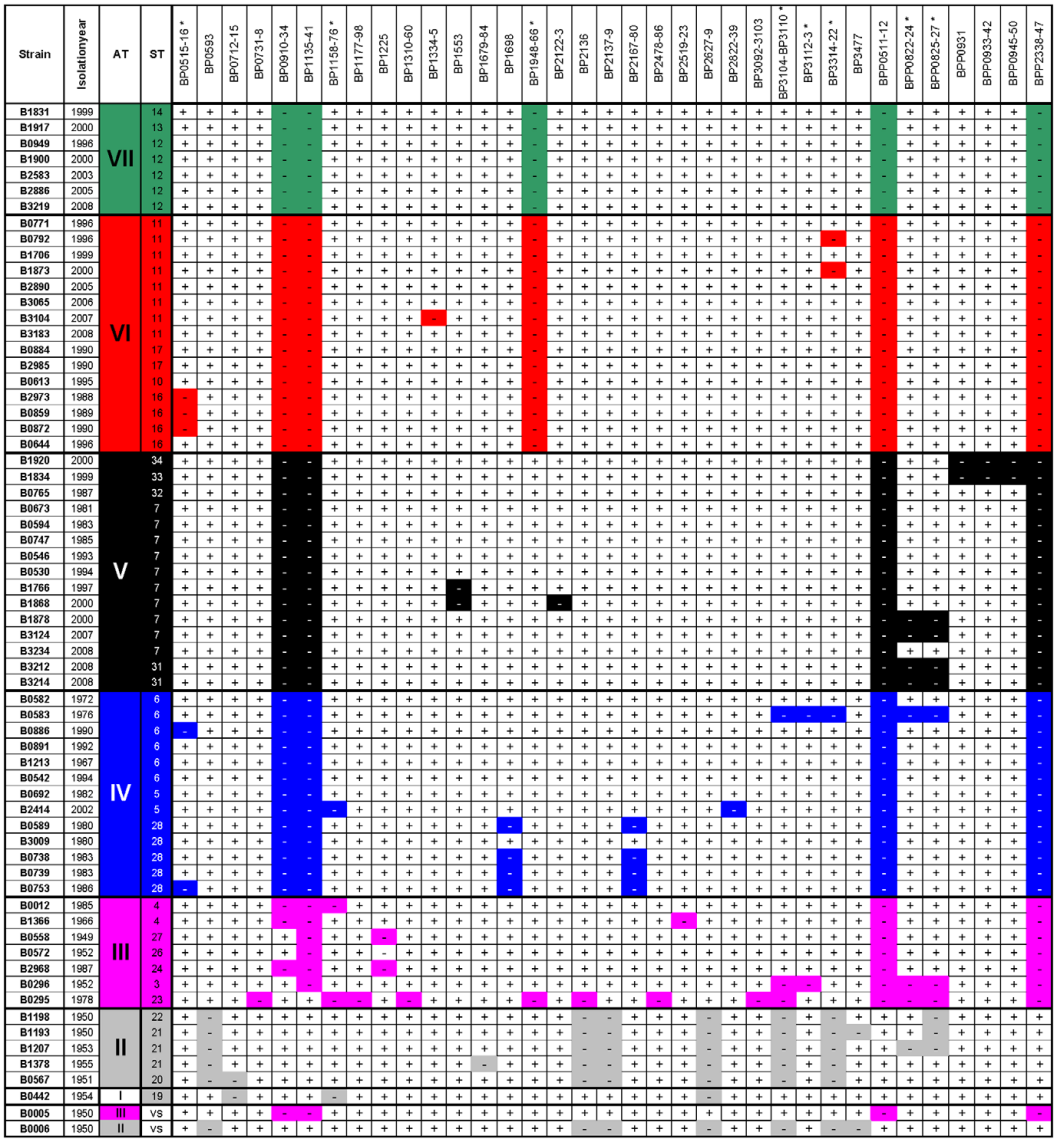
Relationship between phylogeny and gene loss. Strains with their corresponding allele type (AT) and sequence type (ST) are shown in the left columns. Strains were clustered according to their position in the phylogenetic tree as shown in Fig. 1. Colouring of ATs is as in Fig. 1. DNA loci with corresponding gene designations [46] are shown in the top row. The two strains used for the Dutch whole cell vaccine, B0005 and B0006, are indicated in the two bottom rows. Absence and presence of DNA loci are indicated by + and −, respectively and deletions showing homoplasy are indicated with asterisks in the top row. Abbreviations: AT, allele type; ST, sequence type; VS, vaccine strain used for the Dutch WCV.

The genomic content of *B. pertussis* strains is variable due to deletions which arise by homologous recombination between insertion sequence (IS) elements [27], [28], [29], [30], [31], [32]. We investigated whether gene loss could explain the observed shifts in the *B. pertussis* populations. Comparative genomic hybridization (CGH) using microarrays [28], [29] and whole genome sequencing [25], this work] has identified approximately 35 DNA loci which are deleted in one or more of the 65 Dutch strains included in this study (Fig. 4). These 35 deletions resulted in 290 deleted genes in total (Supplementary Table S3). In a previous study, it was remarked that pseudogenes, genes involved in transport and binding and hypothetical genes were overrepresented in deleted regions [28]. Thirty deletions were found in one to ten strains, while ten of these were observed in one strain only. Evidence for homoplasy was seen for eight deletions. As none of these thirty deletions were fixed in the population, they are probably neutral or deleterious.

Five deletions (BP0910–BP0934, BP1135–BP1141, BP1948–BP1966, BPP0511–BPP0512 and BPP2338–BPP2347) were fixed in the population, suggesting that these deletions are neutral or confer a selective advantage. Four out of five deletions (BP0910–BP0934, BP1135–BP1141, BPP0511–BPP0512 and BPP2338–BPP2347) were first found in AT-III, an AT which was found relatively close to the root and predominated in the pre-vaccination era (Fig. 1). The four deletions were absent in one vaccine strain (B0005), but present in the second vaccine strain (B0006). Thus if deletion of these loci reduces the antigenic profile of strains, they may increase fitness in vaccinated populations. Deletions encompassing BPP0511-12 or BPP2338-47 were already fixed in AT-III strains, while deletions encompassing BP0910-34 and BP1135-41 were observed in respectively 43% and 86% of strains belonging to AT-III.

With a single exception, the fifth fixed locus (BP1948–BP1966) was found only in AT-VI and AT-VII. As noted before, this deletion is associated with *ptxP3* strains [29]. The single exception concerns B0295, an atypical *B. pertussis* strain isolated in 1978 which harbours the *ptxP1* allele and contains one of the smallest *B. pertussis* genomes known [28].

In summary, of the deletions fixed in the bacterial population, four (BP0910-34, BP1135-41, BPP0511-12, BPP2338-47) were already present before large shifts were observed, excluding a role in driving these changes. A role for the fifth deletion (BP1948-66) in driving expansion of AT-VI and AT-VII cannot be excluded.

### Comparative genomics

Our results showed that the alleles *ptxA1*, *prn2/3*, *ptxP3* and *fim3-2* were associated with clonal expansions, suggesting that they significantly affect strain fitness. However, we could not exclude the possibility that these alleles were hitchhiking with other, as yet unidentified, alleles which (also) increased fitness and which may be primarily responsible for the selective sweeps. To address this issue, we analyzed the genomes of 11 Dutch clinical isolates, six of which were determined previously [25], and the Tohama I strain [46], representing all ATs except AT-I which was found only once in the Dutch *B. pertussis* populations. We identified 48 SNPs that were linked to the alleles *ptxA1*, *prn2/3*, *ptxP3* and *fim3-2* in these 11 strains (Supplementary Table S4). Using a larger number of strains (N 45) the degree of linkage was further assessed by DNA sequencing (Supplementary Table S3). This revealed that the linkage percentage varied from 89% to 100%. Of these 48 SNPs, 24 were assumed not to affect fitness as they were silent, located in pseudo-genes, in transposons or in intergenic regions downstream from ORFs. The latter SNPs were assumed not to affect expression of ORFs. The seven pseudogenes were initially defined on the basis of the Tohama I sequence [46], but we found that these genes were also inactivated in the two modern isolates B1917 and B1920. A possible role for the remaining 24 SNPs was further explored (Table 1).

**Table 1:** SNPs associated with *ptxA1*, *prn2/3* and *ptxP3*.

SNP no (a)	(putative) product of gene	aa change	subcellular localization (b)	bvg-regulated (c)	linked to	linkage %	remarks (d)
4	serine/threonine protein phosphatase 1 (*pphA*)	F51L	uk	-	ptxA1	100	SNP is located in metallophosphatase domain but not in active site
5	intracellular protease, PfpI family	A142V	cyt	-	ptxA1	98	SNP is not located in conserved site
6	dihydrodipicolinate synthase (*dapA*)	N234D	cyt	-	ptxA1	98	SNP is not located in active sites
7	family-B DNA polymerase	T4M	cyt	-	ptxA1	100	SNP is not located in active site
8	exported protein	L16Q	per	-	ptxA1	98	
10	regulatory lipoprotein	G162S	cyt	rep	ptxA1	100	
13	promoter integral membrane protein/promoter GntR-family transcriptional regulator	intergenic	im/cyt	-	ptxA1	98	
14	UDP-glucose 6-dehydrogenase (*rkpK*)	A60T	uk	-	ptxA1	100	
16	promoter type III secretion system chaparone (*btcA*)/promoter type III secretion system effector (*bteA*)	intergenic	per/es	-/act	prn2/3	100	SNP is located in putative TATA box of btcA
19	endoribonuclease	D62E	cyt	-	prn2/3	100	SNP is not located in putative interaction sites or putative active sites
21	NADH-ubiquinone oxidoreductase, chain N (*nuoN*)	L26F	im	-	prn2/3	100	
22	probable phosphate transporter (*pitA*)	W393.	im	-	prn2/3	96	SNP results in in-frame stopcodon and truncated protein
23	conserved hypothetical protein	T144K	cyt	-	prn2/3	100	
26	promoter exported protein/promoter 30S ribosomal protein S16 (*rpsP*)	intergenic	uk/cyt	-	prn2/3	96	
28	two component system, histidine kinase	A213S	im	act	prn2/3	93	SNP is not located in ATP-binding domain
29	promoter 16S ribosomal RNA	intergenic	cyt	-	prn2/3	93	
32	hydrolase	G138S	uk	-	prn2/3	100	SNP is not located in active sites or conserved positions
33	transport ATP-binding protein	G295S	im	-	prn2/3	100	
35	promoter probable transporter/promoter glycyl-tRNA synthetase alpha chain (*glyQ*)	intergenic	im/cyt	-	ptxP3	96	
37	metal transporter (*gufA*)	V65A	im	-	ptxP3	100	
42	cytoplasmic chaparone torD family protein	N2S	uk	-	ptxP3	96	SNP is not located in active domain
43	type III secretion protein (*bscI*)	Y114C	uk	act	ptxP3	89	SNP is not located in conserved site
44	hypothetical protein	T65I	uk	-	ptxP3	96	
45	promoter integral membrane transport protein	intergenic	im	-	ptxP3	96	

(a) SNP no corresponds to numbers in row 2 of Supplementary table S4: Annotation of SNPs. More information about the SNP can be found in this table.

(b) Subcellular localization was predicted for proteins were no localization was determined experimentally using PSORTb vs 3.0 (http://www.psort.org/psortb/). Cyt: cytoplasm, im: inner membrane, per: periplasm, om: outer membrane, es: extracellular space, uk: unknown, -: not determined.

(c) Information of regulation by bvg was retrieved from Cummings et al. [56], Streefland et al. [78] and de Gouw et al. in preparation. Act: bvg-activated, rep: bvg-repressed.

(d) Information about domains, active sites and conserved positions was derived from SMART (http://smart.embl-heidelberg.de) and Conserved Domain Database (http://www.ncbi.nlm.nih.gov/Structure/cdd/cdd.shtml).

Eight of the 24 SNPs were uniquely associated with *ptxA1*. Affected ORFs coded for proteins located in the cytoplasm or periplasm (N 4 and 1, respectively). The subcellular localization of two proteins could not be predicted. One of the affected ORFs was known to be Bvg-repressed. One SNP was located in an intergenic region between ORFs coding for an integral membrane protein and a GntR-family transcriptional regulator. As the SNP was located in a putative promoter region, it could affect expression of one or both ORFs.

Ten SNPs were uniquely associated with the *prn2/3* alleles. Affected ORFs coded for proteins located in the cytoplasm or inner membrane (N 2 and 4, respectively). The subcellular localization of one protein could not be predicted. One SNP was located in an ORF coding for a two component histidine kinase which was known to be Bvg-activated [56]. Three SNPs were located in putative promoter regions. One of these SNPs could affect transcription of the type III secretion system (TTSS) chaperone and effector genes, *btcA* and *bteA* and was described previously [25], [57]. As the SNP was located in the putative TATA box of *btcA* it might primarily affect transcription of this gene. A second intergenic SNP was located in between two ORFs coding for an exported protein and a 30 s ribosomal protein. The third SNP was located in the putative promoter region of a gene encoding 16S ribosomal DNA.

Six SNPs were uniquely associated with the *ptxP3* allele. Affected ORFs coded for proteins located in the inner membrane (N 2). The subcellular localization of three proteins could not be predicted. Two SNPs were located in putative promoter regions. One of these SNPs could affect transcription of an integral membrane transport protein. A second intergenic SNP was located proximal to the genes for glycyl tRNA synthetase alpha chain (*glyQ*) and a transporter. One SNP was located in an ORF coding for the Bvg-activated type three secretion system protein, BscI.

None of the 24 SNPs were uniquely associated with the *fim3-2* allele.

In summary, the alleles *ptxA1*, *prn2/3*, *ptxP3* were associated with SNPs which could affect both structure and regulation. The SNPs associated with ORFs which are Bvg-activated may be particularly interesting, since many Bvg-activated ORFs have been implicated in host-pathogen interactions [58]. Further functional studies are required to determine if and how these polymorphisms affect strain fitness

## Discussion

Phylogenetic analyses of Dutch strains isolated between 1949 and 2008 revealed a tree, the topology of which was very similar to that of trees derived for the human influenza A virus haemagglutinin genes [59], exhibiting a ladder-like structure with a long trunk and short side branches (Fig. 1). As remarked for the human influenza A virus haemagglutinin tree [59], the trunk corresponds to the progenitor lineage. Mutations that occur along the trunk are eventually fixed, persisting until replaced by subsequent mutations. In contrast, mutations that appear on side branches are eventually lost from the population. The mutations in four virulence-associated genes *ptxP*, *ptxA*, *prn* and *fim3* were found in the trunk of the tree and were fixed until they were replaced by novel mutations in the same gene. When travelling from the root to the tip of the tree, a gradual divergence between the two Dutch whole cell vaccine strains and the *B. pertussis* populations was observed with respect to the four genes. The distribution of ATs in the tree indicated that new genotypes emerged “de novo” rather than being selected from ancient reservoirs, implying a recent change in the *B. pertussis* niche, most likely caused by the introduction of vaccination. These results confirm and substantially extend our previous study in which we showed that *ptxP3* strains arose recently from *ptxP1* strains and spread worldwide [26], [38], [39], [41].

Lan and co-workers used SNP typing to analyze a worldwide collection of 316 *B. pertussis* strains [40], [60]. These authors found a similar relationship between the distribution of mutations in virulence genes and phylogeny and concluded that the observed changes in the *B. pertussis* population were consistent with selection by vaccine-induced immunity. Further, based on SNP typing, six *B. pertussis* clusters were identified of which the most recently evolved showed a worldwide distribution [60]. The latter cluster contained the *ptxP3* strains, in accordance with our previous studies (see above). In line with our observations for Finland and The Netherlands [39], this group provided evidence that *ptxP3* strains were associated with the resurgence of pertussis in Australia [40].

There was a notable linkage between the alleles of the four virulence-associated genes allowing us to define seven allele types (AT-I to AT-VII) which were observed in the Dutch *B. pertussis* population (Fig. 1). A plot of the frequency of these ATs revealed four large shifts in Dutch *B. pertussis* population in the period 1949–2010 (Fig. 2). Each shift was associated with an allele change and resulted largely in the replacement of the extant population by the novel AT (i.e. a clonal sweep). The time which elapsed between the first isolation of a particular AT and when it reached its highest frequency was found to be 19, 16, 12 and 6 years for AT-IV, AT-V, AT-VI and AT-VII, respectively. Comparison of temporal trends in genetic diversity (based on STs) and AT frequencies suggested that point mutations in the four virulence genes were associated with clonal expansions, while mutations in a repeated region of *prn* resulted in a more polyclonal expansion, consistent with the relative frequencies in which these mutations arise. An effect of vaccination on the genetic diversity of *B. pertussis* populations was noted before [38], [52], [54], [61]. These studies were, however, based on IS*1002* fingerprinting and Multiple-Locus Variable Number Tandem Repeat Analysis (MLVA), methods which are less discriminatory than SNP typing. The first shift, in which AT-II and AT-III were replaced by AT-IV, was observed approximately 12 years after introduction of vaccination in 1953. No other obvious relationship between changes in the vaccination programme, such as the introduction of ACVs, and later shifts in ATs were observed. This may be due to the fact that WCVs and ACVs are derived from the same strains. Thus the pertussis antigens in ACVs are identical to those found in WCVs. However, the replacement of the WCV by ACVs was implemented relatively recently in 2005 and it may be too early to expect an effect on the pathogen population structure. ACVs may exert selective pressures that are qualitatively and quantitatively different from WCVs. WCVs induce a Th1 cytokine profile while the response after ACV vaccination shows a mixed Th1/Th2 profile [62]. Further, WCVs induce a broad immune response, with relatively low titers against individual antigens, while ACVs induce an immune response against only a few antigens, but with higher titers. Therefore, the introduction of ACVs may eventually result in new adaptations in the *B. pertussis* population. Indeed, after the introduction of ACVs, in France, Japan and in the Netherlands, strains have been found that do not express pertactin, FHA or pertussis toxin, three components of the currently used ACVs [42], [43], our unpublished data).

Within ATs, we observed large differences in the frequencies of STs. Assuming this was not due to a sampling artefact, this suggests that other, as yet unidentified, polymorphisms may be responsible for fitness differences between ATs and contributed to the clonal sweeps. Such polymorphisms may include horizontally acquired genes, insertions or deletions, chromosomal rearrangements and as yet unidentified SNPs. The sequencing of 12 *B. pertussis* genomes did not provide evidence for recent horizontal acquisition of genes. The technology we used for genome sequencing did not allow the accurate determination of small (∼50 bases) insertions and deletions or large rearrangements. However, large deletions have been identified with CGH [27], [28], [29], [30], [31], [32] and genome sequencing [25], this work]. Gene loss has been suggested to play a role in adaptation of *B. pertussis*. Our phylogenic analysis revealed that of the 35 deletions identified in Dutch strains in previous and this work, five (14%) were fixed in the pathogen population (Fig. 4). Their fixation suggests that these five deletions may have a positive effect on strain fitness. As four of the deletions were already predominantly present in the pre-vaccination era, it seems unlikely that they were the cause for the clonal expansions observed after the introduction of vaccination. The fifth deletion, comprising 18 ORFS (BP1948-66), was linked to the *ptxP3* allele as noted before [29] and may have contributed to the expansion of *ptxP3* strains. The analyses of the deleted ORFs did not provide clues as to how their loss may increase strain fitness.

We used comparative genomics and targeted sequencing to determine if additional SNPs were linked to clonal sweeps. Forty eight SNPs were found with a linkage percentage of ≥89% to the *ptxA1*, *prn2/3* and *ptxP3* or *fim3-2* alleles. After filtering out silent SNPs, SNPs in pseudogenes and SNPs in intergenic regions located downstream of ORFs (hence not involved in transcription initiation), 24 SNPs remained. Of these 24 SNPs, eight, ten and six were associated with, respectively, *ptxA1*, *prn2/3* and *ptxP3* (Table 1). None of the 24 SNPs were linked to *fim3-2*. Only the three SNPs, possibly affecting regulation or structure of Bvg-activated genes will be discussed, as many Bvg-activated proteins have been shown to be involved in host-pathogen interactions [58]. One SNP, associated with *prn2/3* could affect transcription of the type III secretion system (TTSS) chaperone and effector genes, *btcA* and *bteA*. For *B.bronchiseptica* it has been shown that BteA is translocated into the host cell and is cytotoxic for a wide range of mammalian cells [63], [64]. This SNP, identified previously [25], [57], was also observed in five *prn2* strains, but not in five *prn1* strains, isolated in Japan [57]. Interestingly, in the Japanese study it was found that *bteA* was expressed at a higher level in the *prn2* strains compared to the *prn1* strains. The lower level of expression in *prn1* strains was associated with an IS insertion upstream of *bteA*. It was suggested that the higher expression of *bteA* in *prn2* strains may have contributed to its emergence in Japan. An IS element upstream of *bteA* has not been found in the sequenced genome of other *prn1* strains [25], [46], [65]. We are currently investigating the effect of the TTSS-SNP on expression. The second SNP was linked to *ptxP3*, located in *bscI* and resulted in an amino acid change. The *bscI* gene is part of a large TTSS cluster, *bsc*, which is Bvg activated and is involved in secretion of proteins directly into host cells [66], [67]. The homolog of *bscI* in *Yersinia* is essential for secretion of proteins [67]. The allele was designated *bscI2*. The linkage between *bscI2* and *ptxP3* was not absolute and found in nine out of the 14 *ptxP3* strains investigated. Therefore this SNP cannot be responsible for the early phase of the clonal expansion of *ptxP3* strains. However if this SNP has a positive effect on the strain fitness it may contribute to a further expansion of strains with the *ptxP3* allele.

The third SNP was located in a histidine kinase of a two component sensory transduction system and is partially linked to *prn2/3*. Of the investigated strains 3 out of 12 *prn1* strains and all *prn2/3* strains have this SNP. This SNP may have contributed to the expansion of *prn2/3* strains as two component sensory transduction systems are important in adapting to environmental stimuli. Indeed, the major regulator of virulence genes in *B. pertussis*, BvgAS, belongs to this category of regulatory systems [68]. Although the remaining SNPs are not located in (known) virulence associated genes, this does not exclude a role in adaptation. Further studies of the identified loci may elucidate the role of these polymorphisms in the ecology of *B. pertussis*.

Several lines of evidence support a role for the *ptxA*, *prn2/3*, *ptxP3* and *fim3-2* alleles in driving the observed clonal sweeps, although the above described, or as yet to be identified polymorphisms, may also have played a role. All mutations in ORFs that caused transitions between alleles resulted in amino acid changes. Further, the polymorphic region which distinguishes Prn1, Prn2 and Prn3 has been shown to be part of a B-cell epitope [37], [69], and variation in this region was shown to affect vaccine efficacy in a mouse model and antibody binding [37], [70]. The variable amino acid which distinguishes Fim3-1 and Fim3-2 is part of a seven residue long peptide recognized by human sera [71]. Significantly, the corresponding codon is also polymorphic in *B. bronchiseptica* (Supplementary Fig. S1). *B. pertussis* fimbrial genes are subject to phase variation and strains may contain silent *fim3* genes [55]. If the switch from *fim3-1* to *fim3-2* has driven a clonal sweep, one would expect *fim3-2* to be mainly associated with strains expressing *fim3*. This is indeed what we found, as 99% of the *fim3-2* strains produced Fim3 fimbriae. It is plausible that increased Ptx production, associated with the *ptxP3* allele has also driven a clonal sweep. Ptx has been shown to suppress both innate and acquired immunity in mice [72], [73], [74], [75], [76] and increased production may be particularly important in a population primed by vaccination [39]. We have found that polymorphism in *ptxP* affects colonization of mice [77]. It is likely that the mutations have a cumulative effect. In a series of elegant experiments with isogenic strains differing only in *ptxA* or *prn*, Komatsu and co-workers [33] showed that mismatches with vaccine strains in these genes reduced vaccine efficacy in a mouse model. Interestingly, a mismatch in both genes was required to have a measurable effect in vivo.

In conclusion, our work provides evidence that *B. pertussis* has adapted by the accumulation of small mutations. It seems that, even in the context of complex bacterial genomes, small mutations in single genes can have a significant effect on strain fitness, resulting in clonal sweeps within a period of six to 19 years. As illustrated by the emergence of *ptxP3* strains, the effect may be large enough to be of relevance for public health [39], [40]. We have identified several novel SNPs which may have contributed to the adaptation of *B. pertussis*. Clarification of the role of these SNPs may elucidate further how *B. pertussis* has persisted and resurged in the phase of intensive vaccination, and ultimately allow us to improve pertussis vaccines on a rational basis [9], [39].

### Accession numbers

The sequence data of the five strains used for whole genome sequencing, B0296, B0400, B0496, B0738 and B3405, were submitted to the Sequence Read Archive (http://www.ncbi.nlm.nih.gov/sra) under accession number SRA051375. The nucleotide sequence of the *fim3-5* allele has been deposited in GenBank (http://www.ncbi.nlm.nih.gov/nuccore) under accession number CAA52217.

## Supporting Information

Figure S1 **Variation in the Dutch** ***B. pertussis*** **populations in the genes for the pertussis toxin promoter (** ***ptxP*** **), the pertussis toxin A subunit (** ***ptxA*** **), fimbrial subunit 2 (** ***fim2*** **), fimbrial subunit 3 (** ***fim3*** **) and pertactin (** ***prn*** **).** Dots and dashes indicate identity and gaps, respectively. Positions with silent mutations in *prn* are shaded. The initiation codon for *ptxA* has been underlined in the *ptxP* sequence. Numbering of the nucleotides in *ptxA*, *fim3*, *fim2* and *prn* is relative to the start of the open reading frame. In the *prn* sequences, the three types of repeated sequences and the RGD sequence, involved in attachment to mammalian cells, are underlined [6], [23], [44]. Allele *fim3-5*, which is found in *B. bronchiseptica* is also indicated.
(TIF)

Table S1 **Characteristics of 704** ***Bordetella pertussis*** **strains used in this study.** Strains were isolated in the period 1949–2010. *PtxP*, *ptxA*, *prn* and *fim3* alleles were determined for all strains. The *fim2* allele was determined for 272 strains. SNP typing was performed for 198 strains and concatenated SNP sequences are indicated.
(XLS)

Table S2 **85 SNPs used in this study.** The locus containing the SNP, the position of the SNP in the ORF and the reference position in Tohama I are indicated. Abbreviation: SNP, single nucleotide polymorphism.
(XLS)

Table S3 **List of all deleted genes.** RD number, locus, name and gene product are indicated.
(XLS)

Table S4 **SNPs linked to the alleles** ***ptxA1***, ***prn2/3*** **,** ***ptxP3*** **and** ***fim3-2*** **.** SNPs were identified by comparing the genome sequences of 11 *B. pertussis* clinical isolates and the Tohama I strain [46] [25] and this work]. The degree of linkages was further assessed using a larger number of strains (N 45). Strains were clustered according to their position in the phylogenetic tree as shown in Fig. 1. Colouring of ATs is as in Fig. 1. Abbreviations: AT, allele type; ST, sequence type.
(XLS)
