# Supplementary figures and images for: Small Mutations in Bordetella pertussis Are Associated with Selective Sweeps
Source: PLoS One. 2012 Sep 28;7(9):e46407. doi: 10.1371/journal.pone.0046407 (PMC3460923; doi:10.1371/journal.pone.0046407)

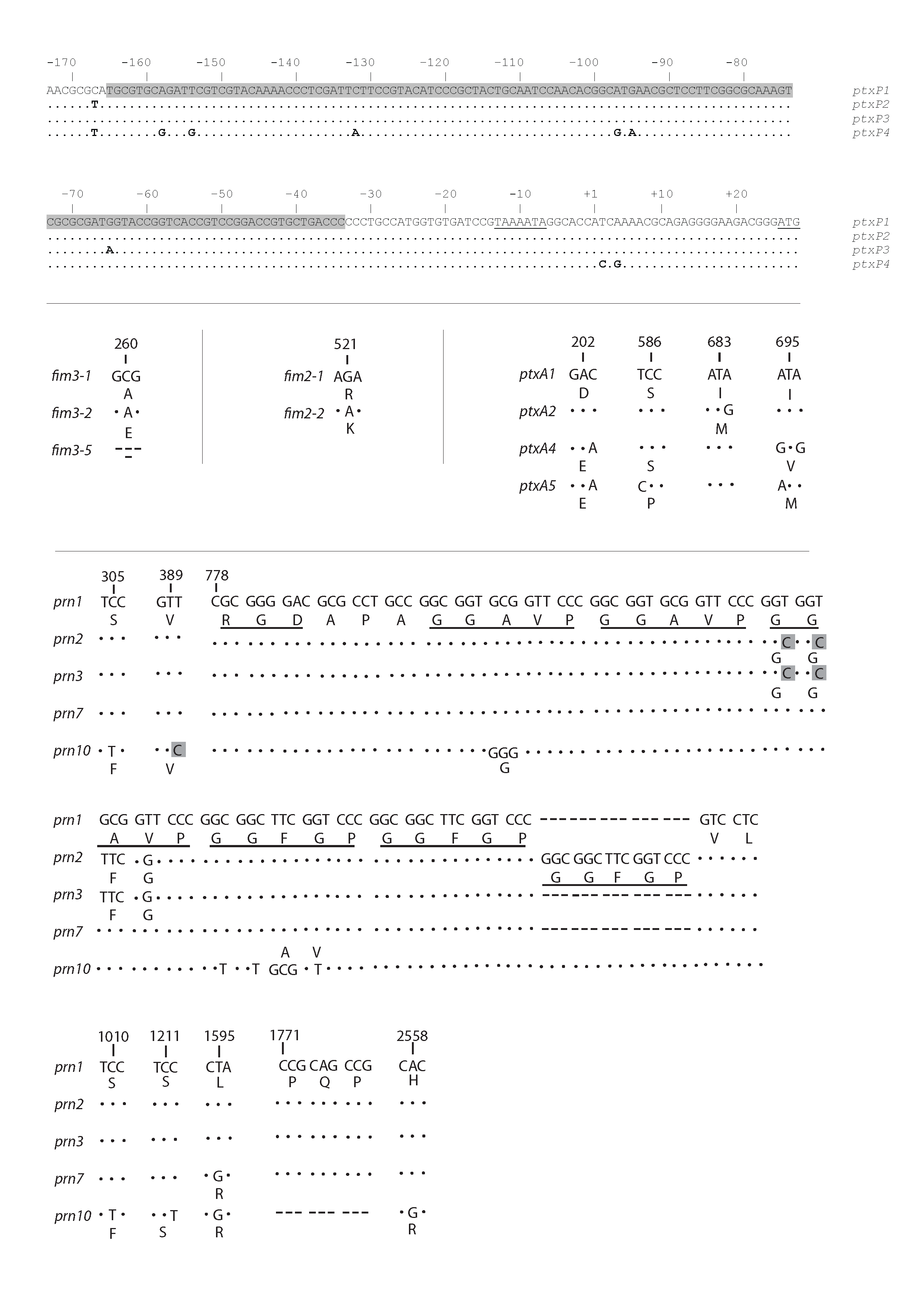

Supplement: Figure S1 — Variation in the Dutch B. pertussis populations in the genes for the pertussis toxin promoter ( ptxP ), the pertussis toxin A subunit ( ptxA ), fimbrial subunit 2 ( fim2 ), fimbrial subunit 3 ( fim3 ) and pertactin ( prn ). Dots and dashes indicate identity and gaps, respectively. Positions with silent mutations in prn are shaded. The initiation codon for ptxA has been underlined in the ptxP sequence. Numbering of the nucleotides in ptxA, fim3, fim2 and prn is relative to the start of the open reading frame. In the prn sequences, the three types of repeated sequences and the RGD sequence, involved in attachment to mammalian cells, are underlined [6], [23], [44]. Allele fim3-5, which is found in B. bronchiseptica is also indicated. (TIF) [file pone.0046407.s001.tif]
